# ATRA Inhibits the Proliferation of DU145 Prostate Cancer Cells through Reducing the Methylation Level of HOXB13 Gene

**DOI:** 10.1371/journal.pone.0040943

**Published:** 2012-07-13

**Authors:** Zhiwei Liu, Guoling Ren, Chenyan Shangguan, Lijing Guo, Zhixiong Dong, Yueyang Li, Weina Zhang, Li Zhao, Pingfu Hou, Yu Zhang, Xiuli Wang, Jun Lu, Baiqu Huang

**Affiliations:** 1 The Institute of Genetics and Cytology, Northeast Normal University, Changchun, China; 2 College of Life Sciences, Daqing Normal University, Daqing, Heilongjiang, China; 3 The College of Life Science, Tianjin Normal University, Tianjin, China; 4 The Key Laboratory of Molecular Epigenetics of Ministry of Education, Northeast Normal University, Changchun, China; Wayne State University School of Medicine, United States of America

## Abstract

All-trans retinoic acid (ATRA) has been widely investigated for treatments of many cancers including prostate cancer. *HOXB13*, silenced in androgen receptor-negative (AR^−^) prostate cancer cells, plays a role in AR^−^ prostate cancer cell growth arrest. In this study we intended to elucidate the mechanisms that are involved in the proliferation inhibition of AR^−^ prostate cancer cells triggered by ATRA. We discovered that ATRA was able to induce the growth arrest and to increase *HOXB13* expression in AR^−^ prostate cancer cells. Both EZH2 and DNMT3b participated in the repression of *HOXB13* expression through an epigenetic mechanism involving DNA and histone methylation modifications. Specifically, EZH2 recruited DNMT3b to *HOXB13* promoter to form a repression complex. Moreover, ATRA could upregulate *HOXB13* through decreasing EZH2 and DNMT3b expressions and reducing their interactions with the *HOXB13* promoter. Concurrently, the methylation level of the *HOXB13* promoter was reduced upon the treatment of ATRA. Results from this study implicated a novel effect of ATRA in inhibition of the growth of AR^−^ resistant human prostate cancer cells through alteration of *HOXB13* expression as a result of epigenetic modifications.

## Introduction

Prostate cancer is the most common malignancy in men in Europe, North America and in a number of African countries [1], [2]. The incidence of prostate cancer has increased following the aging of population worldwide [3]. At present, conventional therapies such as surgery and hormone treatment frequently fail to achieve the satisfactory effect. Moreover, cancer cells may acquire the hormone and drug resistant features under these treatment stresses [4]. So far, efforts to conquer this malignant disease have achieved limited success [5]. Thus, researches aimed at the development of new and more effective therapeutic strategies for prostate cancer remain an open opportunity.

*HOXB13* gene belongs to a large homeobox superfamily, many of which are transcription factors that regulate axial regional specification during embryonic development [6], [7]. Limited expression of *HOXB13* was observed at the caudal extent of the spinal cord, urogenital sinus, and colon and rectum cells in an androgen-independent manner; but it expressed in prostate with remarkable tissue-specificity to maintain its normal physiological function and to induce the terminal differentiation [8], [9]. *HOXB13* is silenced in androgen receptor-negative (AR^−^) prostate cancer cells. Jung *et al*. [5] showed that ectopic expression of *HOXB13* in a prostate cancer cell line induced G1 cell cycle arrest through negative regulation of T-cell factor-4, but did not lead to change in apoptotic rate. Overexpression of *HOXB13* in AR^−^ prostate cancer cells resulted in significant inhibition of cell growth [10]. However, the mechanism underlying this gene silencing is not fully understood. Recently, we investigated the functions of polycomb group (PcG) proteins and their epigenetic actions in silencing of *HOXB13* in prostate cancer cells, and found that there was a crosstalk between histone acetylation and members of PcG proteins on repressing the *HOXB13* expression [11]. In this study, we provided further evidence that DNA methyltransferases (DNMTs) and PcG proteins synergistically inhibited *HOXB13* promoter activity.

All-trans retinoic acid (ATRA), the vitamin A metabolite, plays an essential role in the development by regulating cellular processes such as proliferation, differentiation and migration [12]. ATRA implements its effect by binding specific nuclear receptor superfamily, the retinoic acid receptors (RARs). The RARs form a heterodimer with the retinoid X-receptor [13]. Earlier studies revealed that treatment of leukemic cells with ATRA resulted in the apoptosis, presumably secondary to the differentiation process [14], [15]. Since ATRA can rectify aberrant cell growth and induce apoptosis, it has been widely investigated in preclinical and clinical trials for the treatment of many cancer types, including early gastric cancer and prostate cancer [16], [17]. In AR^−^ and drug resistant DU145 prostate cancer cells, ATRA was demonstrated to increase the sensitivity of cells to anticancer agent docetaxel; however the mechanisms how ATRA alone induces cell growth arrest remain unclear [18].

PcG proteins are global repressors of gene expression through the formation of polycomb repressive complex (PRC), such as PRC1 and PRC2 [19]. Several PcG proteins have been implicated in oncogenic activities [20]. There have been indications that PcG repressor activity is increased during prostate cancer progression [21]. Moreover, some *PcG* gene products were also found to be required for the stable silencing of *Hox* genes throughout *Drosophila* development [22]. Enhancer of Zeste Homologue 2 (EZH2), the catalytic subunit of PRC2, possesses a histone methyltransferase activity for histone 3 lysine 27 trimethylation (H3K27me3), which establishes a strong repressive signal for gene expression [19]. It was shown that ectopic overexpression of *EZH2* not only stimulated cell proliferation, but also promoted anchorage-independent growth and cell invasion *in vitro* [20]. In contrast, depletion of *EZH2* by small interfering RNAs (siRNAs) inhibited cell proliferation and induced apoptosis in prostate, breast, and colon cancer cells [23], [24], [25].

Methylation of DNA is a major epigenetic modification that influences gene transcription. DNA methylation in mammalian cells is established and maintained by DNMTs. Methylation is initiated by highly homologous DNMT3a and DNMT3b, and heritably propagated by DNMT1 [26]. Among these three enzymes, upregulation of *DNMT3b* is a characteristic of many cancer cells, and DNMT3b may play a causal role in tumorigenesis [27]. Studies in a mouse model showed that overexpression of *DNMT3b*, but not *DNMT3a*, promoted colon tumorigenesis in *ApcMin/+* mice [28]. A previous study demonstrated that expression of *HOXB13* was controlled in a methylation-dependent manner and its methylation was correlated positively with tumor grade and microvessel invasion [29]. In colon cancer cells, *HOXB13*, as a target of DNMT3b, was methylated at an upstream CpG island, and functioned as a tumor suppressor in primary colorectal tumors [26].

Since DNMT3b does not have DNA-binding domains, it needs transcription co-factors for interacting with specific gene sequences [30]. It has been known that many members of PcG, such as EZH2, possess the binding domains for *HOXB13* promoter, and they suppress *HOXB13* expression through histone methylation [31]. Significantly, EZH2 has been implicated to play a key role in mediating both histone methylation and DNA methylation of *HOXB13* gene in some cancer cells [21], [32]. Also, we previously reported that YY1 and the histone deacetylase 4 (HDAC4) affected prostate cancer cell growth by repressing *HOXB13* transcription through histone modification [11]. These available data intrigued us to speculate that EZH2 might be able to recruit DNMT3b to *HOXB13* promoter to repress its expression through particular epigenetic modifications.

The purpose of this study was to elucidate the mechanisms that are involved in the inhibition of proliferation in AR^−^ prostate cancer cells triggered by ATRA. Our results revealed that ATRA inhibited the growth of DU145 prostate cancer cells through upregulating *HOXB13* expression by reducing its methylation level. This was achieved by ATRA to impair the action of EZH2 and DNMT3b, which are responsible for the methylation of *HOXB13* promoter. Data unraveled from this study may provide useful clues to the development of new therapeutic strategies for AR^−^ prostate cancer that involve the use of ATRA and epigenetic modifiers.

## Results

### HOXB13 was Involved in ATRA-induced DU145 Cell Growth Arrest

**Figure 1:**
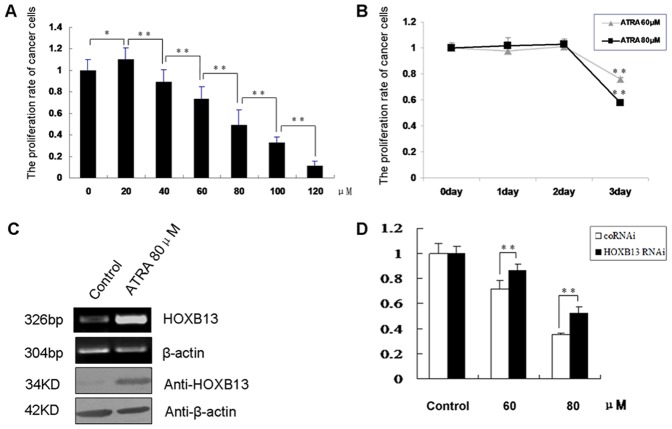
ATRA induced DU145 cell growth arrest through upregulation of HOXB13. (**A**) Dose-dependent effect of ATRA on DU145 cell growth arrest. DU145 cells were treated with various concentrations of ATRA for 72 h. *P<0.05, **P<0.01 (n = 6). (**B**) Time course of the growth arrest effect of ATRA in DU145 cells. *P<0.05, **P<0.01 (n = 6). (**C**) ATRA increased HOXB13 expression in DU145 cells. DU145 cells were treated with 80 µM of ATRA for 72 h. Total RNA was extracted for RT-PCR (*uppe*r), and the whole cell lysates was prepared for western blotting (*lower*). (**D**) The ATRA-induced DU145 cell growth arrest was partially reversed by suppression of HOXB13 expression. The cell growth potent was measured by MTT assays. *P<0.05, **P<0.01 (n = 6).

ATRA was reported to be able to induce cell growth arrest, but the mechanism is not fully clear. Overexpression of HOXB13 in AR^−^ prostate cancer cells can also result in significant inhibition of cell growth. To clarify whether HOXB13 plays a role in ATRA-induced growth arrest in prostate cancer cells, we first tested the relative cell survival rate upon the treatment of ATRA. DU145 cells were exposed to increasing concentrations of ATRA (from 20 to 120 µM) for 24, 48 and 72 h. As evidenced in Fig. 1A and 1B, ATRA indeed induced cell growth arrest in DU145 cells in a dose-dependent manner. High cytotoxicity was observed at 72 h. Meanwhile, both HOXB13 mRNA and protein levels were elevated in DU145 cells upon ATRA treatment (Fig. 1C). We further verified the effect of ATRA in another AR^−^ prostate cancer cell line PC-3, and we observed the similar dose-dependent effect of ATRA on cell growth arrest, as revealed by MTT assays (Fig. S1). Meanwhile, both HOXB13 mRNA and protein levels were also elevated upon ATRA treatment (Fig. S2).

Next, to test the role of HOXB13 in ATRA-mediated DU145 cell growth arrest, we transiently transfected the *HOXB13* siRNA plasmid into DU145 cells treated with ATRA. The construction and interfering efficiency of the HOXB13 siRNA were described in our previous report [11]. We found that knockdown of HOXB13 by siRNA apparently reversed the cell growth arrest induced by ATRA (Fig. 1D). This implicated that the ATRA-induced DU145 cell growth arrest was associated, at least in part, with the expression of HOXB13.

### DNMT3b Participated in HOXB13 Transcriptional Repression

**Figure 2:**
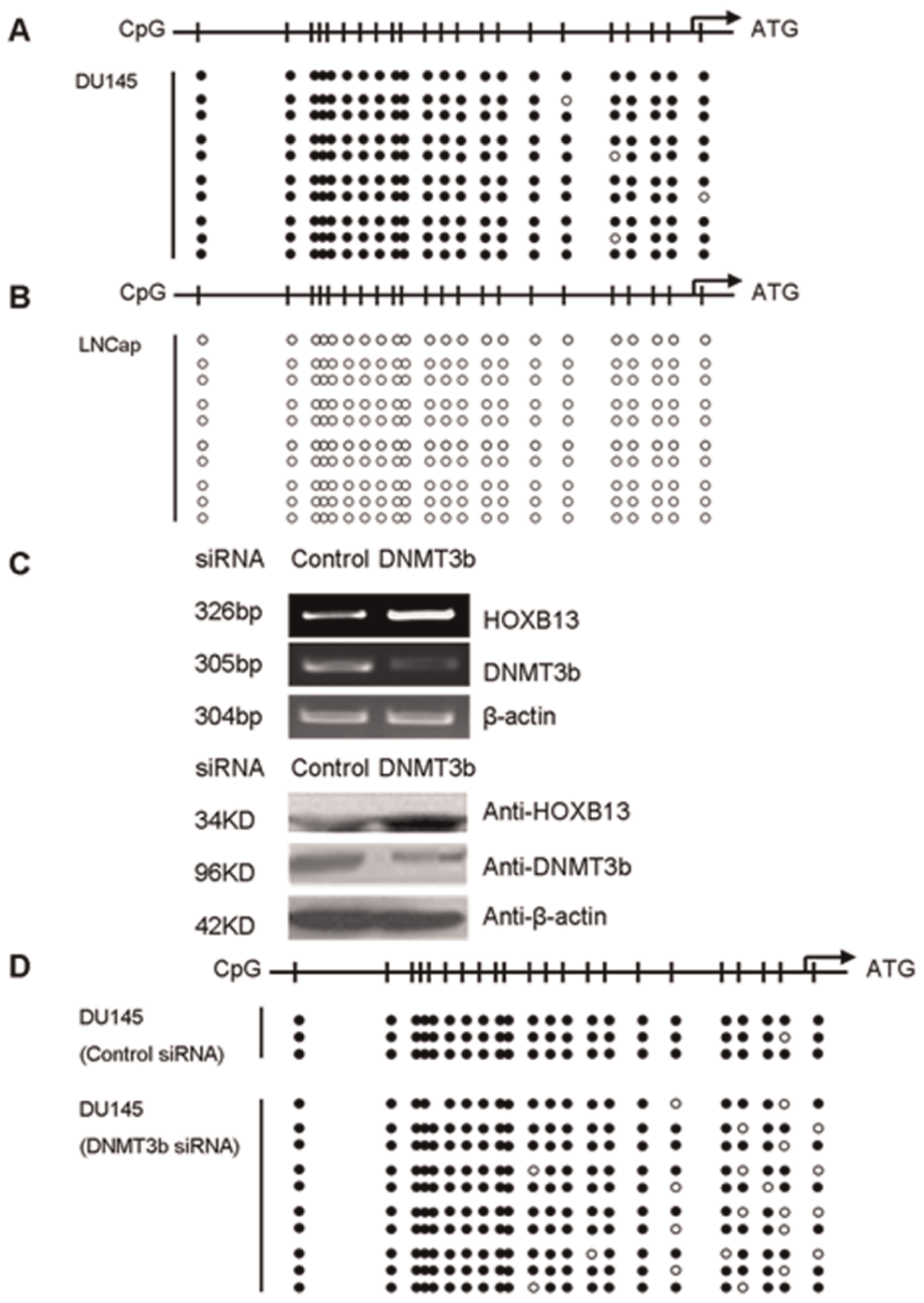
HOXB13 expression was regulated by DNA methylation. BSP assays showed the methylation of CpG islands at the HOXB13 promoter in highly malignant DU145 cells (**A**) and in lowly malignant LNCap cells (**B**). (**C**) HOXB13 was upregulated in DU145 cells transfected with the DNMT3b siRNA plasmid, compared with the cells transfected with control siRNA vector. (**D**) BSP assays showed the reduced methylation level of HOXB13 promoter in DU145 cells transfected with DNMT3b siRNA (*lower*), compared with control siRNA (*upper*).

*HOXB13* gene is silenced in AR^−^ prostate cancer cells and overexpression of HOXB13 in AR^−^ prostate cancer cells resulted in significant inhibition of cell growth [10]. We then investigated the molecular events that are involved in *HOXB13* gene silencing in AR^−^ prostate cancer cells. The promoter of *HOXB13* has been frequently reported to be hypermethylated in various cancers [33]. To clarify whether silencing of *HOXB13* gene is mediated by DNA hypermethylation in AR^−^ prostate cancer cells, we assessed the methylation status of the *HOXB13* promoter by using the bisulfite-sequencing PCR (BSP), and we found that the *HOXB13* promoter was more intensively methylated in DU145 cells in comparison with that in the lowly malignant LNCap cells (Fig. 2A, B).

Since the DNA methyltransferase DNMT3b plays an important role in methylation of cancer-related genes, and *HOXB13* was reported to be a target gene of DNMT3b in primary colorectal tumors [26], [27], we speculated that DNMT3b may also be involved in *HOXB13* transcriptional repression in DU145 cells. To verify this assumption, we knocked down the DNMT3b expression with a specific siRNA, and we found that the HOXB13 expression was apparently increased in DU145 cells and the interfering efficiency of the DNMT3b siRNA was shown (Fig. 2C). The results from BSP assays showed that the methylation level of *HOXB13* promoter in DU145 was reduced after knockdown of the endogenous DNMT3b (Fig. 2D, lower), compared with the control cells (Fig. 2D upper). These results suggested that the DNA methylation modification mediated by DNMT3b was responsible for the silencing of *HOXB13* in DU145 cells.

### The PcG Proteins EZH2 and BMI1 Participated in HOXB13 Transcriptional Repression

**Figure 3:**
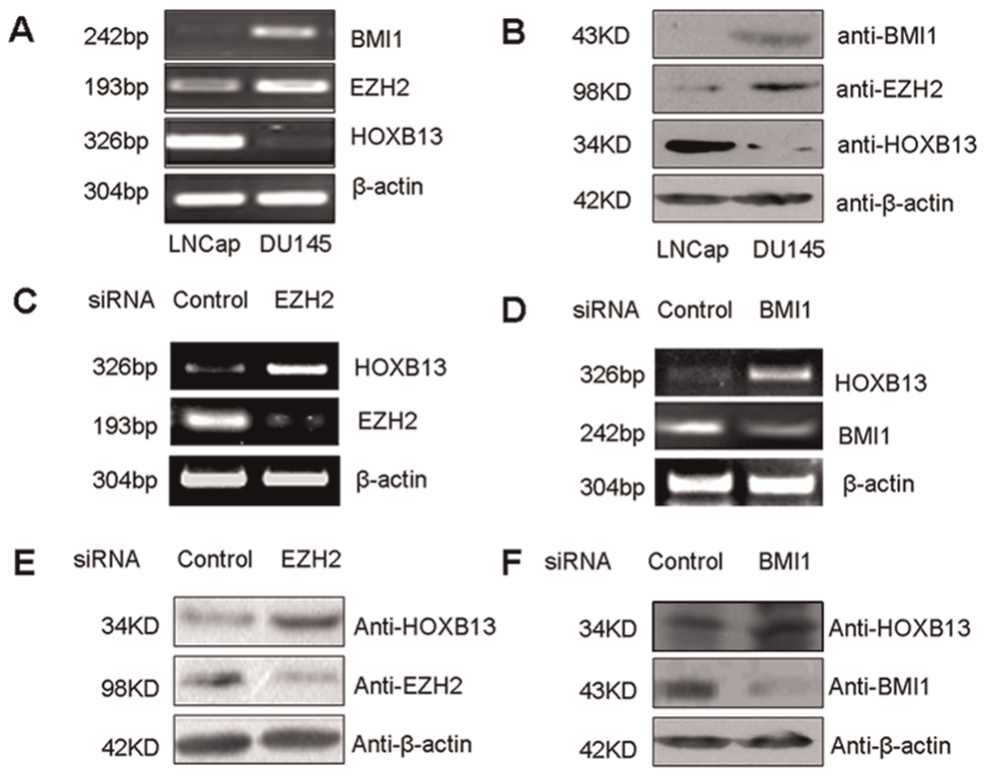
Polycomb group (PcG) proteins EZH2 and BMI1 participated in HOXB13 transcriptional repression. The mRNA (**A**) and protein (**B**) levels of BMI1, EZH2 and HOXB13 in different malignancy prostate cancer cells, LNCap and DU145. (**C**) The mRNA levels of HOXB13 and EZH2 in DU145 cells transfected with EZH2 siRNA plasmid. (**D**) The mRNA levels of HOXB13 and BMI1 in DU145 cells transfected with BMI1 siRNA plasmid. (**E**) The protein levels of HOXB13 and EZH2 in DU145 cells transfected with EZH2 siRNA plasmid. (**F**) The protein levels of HOXB13 and BMI1 in DU145 cells transfected with BMI1 siRNA plasmid.

Next, we intended to determine the transcription factor(s) that may participate in regulation of *HOXB13* gene. The PcG proteins have been reported to play a role in progression of prostate and breast cancers, as well as in silencing of *HOXs* [3], [21], [34], [35]. We first determined that expressions of PcG proteins EZH2 and BMI1 were significantly higher in highly malignant DU145 cells than in lowly malignant LNCap cells, and their expressions were negatively correlated to HOXB13 expression, as revealed by RT-PCR and western blotting (Fig. 3A, B).

To further clarify whether EZH2 and BMI1 participated in *HOXB13* transcriptional repression in DU145 prostate cancer cells, we constructed EZH2 siRNA and BMI1 siRNA plasmids and transfected them into DU145 cells, respectively. We detected that both HOXB13 mRNA (Fig. 3C, E) and protein (Fig. 3D, F) levels were upregulated when EZH2 and BMI1 were knocked down. The inhibitory efficiencies of these siRNA plasmids on endogenous EZH2 and BMI1 at mRNA and protein levels were shown (Fig.3C, D and E, F). These results indicated that EZH2 and BMI1 may repress HOXB13 expression by mediating the histone methylation modification.

### DNMT3b was Recruited to HOXB13 Promoter by EZH2

**Figure 4:**
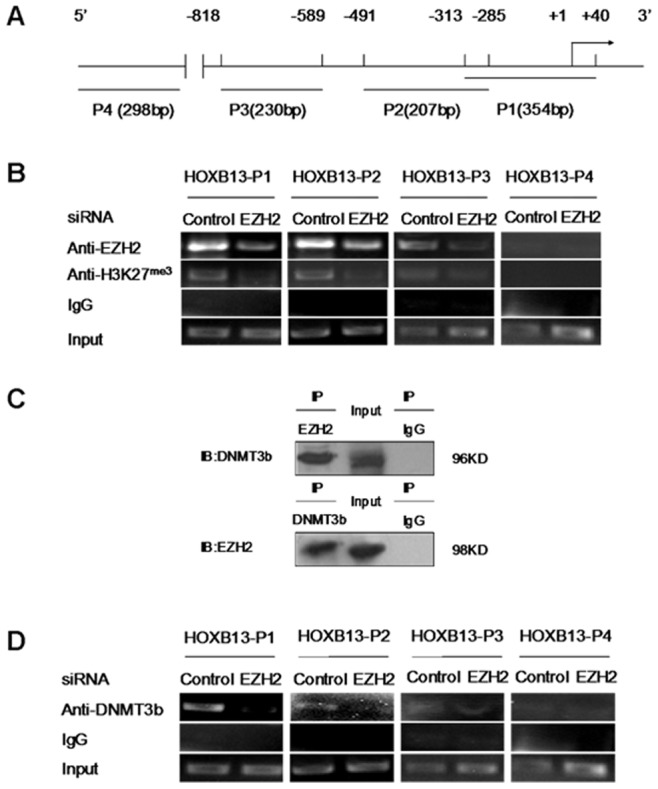
DNMT3b was recruited to HOXB13 gene promoter by EZH2. (**A**) Diagram of the 5′-ﬂanking region of HOXB13 gene. P1, P2 and P3 denote the three promoter regions of HOXB13 gene analyzed in ChIP assays, and P4 is a distal negative control. (**B**) ChIP assays in DU145 cells transfected with EZH2 siRNA and immunoprecipitated with anti-EZH2 antibody and anti-H3K27me3 antibody, respectively. (**C**) CoIP assays for detecting the association of DNMT3b with EZH2 in DU145 cells. Cell nuclear extracts were prepared and precipitated with anti-EZH2 or anti-DNMT3b antibody, and detected by immunoblotting with anti-DNMT3b or anti-EZH2 antibody. (**D**) ChIP assays were performed in DU145 cells transfected with EZH2 siRNA and DNA was immunoprecipitated with anti-DNMT3b antibody. The amounts of precipitated DNA were determined by PCR.

As co-repressors of transcription, DNMTs do not possess canonical DNA-binding domains and they are usually recruited to specific chromatin regions by transcription factors [26], [30]. Significantly, EZH2 has been reported to play a key role in mediating both histone methylation and DNA methylation of *HOXB13* gene in some cancer cells [21], [32]. We then wanted to determine whether EZH2 recruits DNMT3b to *HOXB13* promoter. We performed ChIP assays to detect changes in association of EZH2 at the three defined regions (P1–P3) of the *HOXB13* promoter upon the knockdown of endogenous EZH2. The far upstream P4 region was chosen as a negative control (Fig. 4A). The ChIP results demonstrated that the abundance of EZH2 at P1–P3 of *HOXB13* promoter was reduced when endogenous EZH2 was suppressed by *EZH2* siRNA in DU145 cells. Concurrently, the H3K27me3 level associated with EZH2 function at P1–P3 was apparently reduced (Fig. 4B).

Moreover, CoIP assays with DU145 cell extracts revealed that complexes were immunoprecipitated by anti-DNMT3b or anti-EZH2 antibodies, and could be detected in immunoblotting by anti-EZH2 or anti-DNMT3b antibodies, respectively (Fig. 4C), suggesting that DNMT3b and EZH2 were present in the same complex. Our ChIP data in Fig. 4D further indicated that the abundance of DNMT3b at the three promoter regions (P1–P3) was reduced upon the knockdown of endogenous EZH2. Thus, these results provided evidence that DNMT3b was recruited to *HOXB13* promoter by EZH2.

### ATRA Impaired EZH2 and DNMT3b Expression and Weakened their Interactions with HOXB13 Promoter

**Figure 5:**
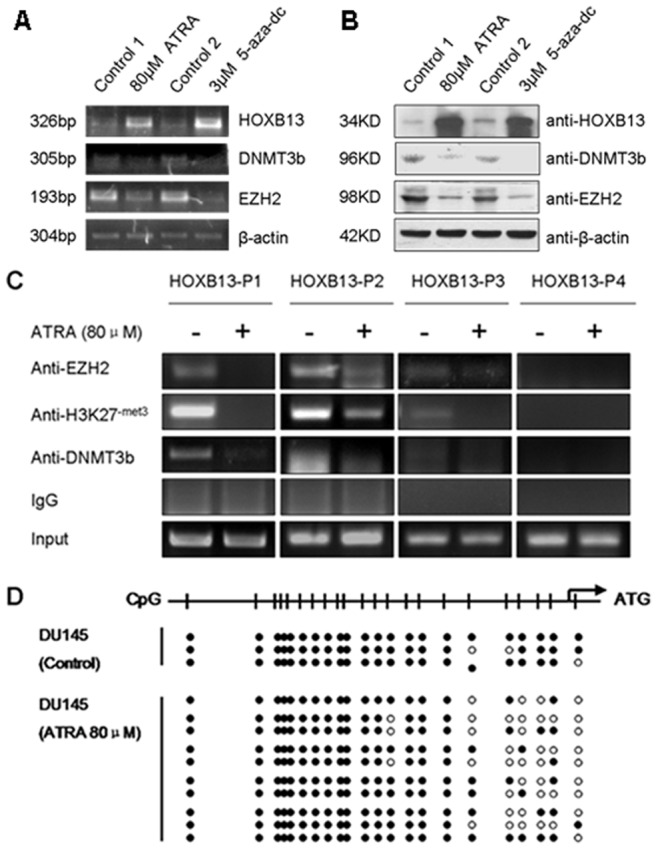
ATRA upregulated HOXB13 expression by inhibiting EZH2 and DNMT3b in DU145 cells. Treatment of 80 µM of ATRA upregulated HOXB13 and downregulated EZH2 and DNMT3b expressions at mRNA level (**A**) and protein level (**B**) in DU145 cells. 5-aza-dc was used as a positive demethylation control. Control 1 was isopycnic absolute ethanol and control 2 was isopycnic acetic acid (**C**) ATRA impaired the bindings of EZH2 and DNMT3b, and reduced the H3K27me3 level at HOXB13 promoter. ChIP assays were performed in DU145 cells treated with 80 µM ATRA and immunoprecipitated with anti-H3K27me3, anti-DNMT3b or anti-EZH2 antibody. The amounts of precipitated endogenous HOXB13 promoter DNA were determined by PCR. (**D**) BSP assays showed the reduced methylation of HOXB13 promoter in DU145 cells after treatment upon 80 µM ATRA, compared with the untreated control.

We have demonstrated above that HOXB13 was downregulated by EZH2 and DNMT3b, and ATRA treatment resulted in a raise in *HOXB13* expression in DU145 cells. To verify our assumption that ATRA may facilitate HOXB13 expression through impairing the negative regulatory effects of EZH2 and DNMT3b on HOXB13, we examined the EZH2 and DNMT3b expression in DU145 cells treated with 80 µM ATRA, and 5-aza-2′-deoxycytidine (5-aza-dc) was used as the demethylation control. The results showed that expressions of both EZH2 and DNMT3b were downregulated upon ATRA treatment at both mRNA and protein levels (Fig. 5A, B). Similar results were obtained from a parallel study using another AR^−^ malignant prostate cancer cell line PC-3 (Fig. S2).

Our ChIP assays further demonstrated that the bindings of endogenous EZH2 and DNMT3b on *HOXB13* promoter was reduced after ATRA treatment, and meanwhile the H3K27me3 levels at P1–P3 regions were apparently decreased (Fig. 5C).

Moreover, BSP assays revealed that treatment with ATRA resulted in the reduction of methylation level of CpG islands on *HOXB13* promoter in contrast to that of untreated DU145 cells (Fig. 5D). Apparently, EZH2 recruited DNMT3b to *HOXB13* promoter and triggered the gene silence by inducing the H3K27me3 and by increasing the methylation of CpG islands. Meanwhile, both the DNA methylation and H3K27me3 of *HOXB13* promoter could be reduced by ATRA.

## Discussion

ATRA plays an essential role in the development by regulating various cellular processes and it has been widely investigated in preclinical and clinical trials as an agent for the treatment of many cancer types, including early gastric cancer and prostate cancer [16], [17]. Also, HOXB13 was shown to play a part in growth arrest in AR^−^ prostate [10], colorectal cancer [26] and renal cell carcinoma [29]. In line with these previous data, results from this study demonstrated that ATRA was able to induce growth arrest of two AR^−^ prostate cancer cells DU145 and PC-3 in a dose-dependent manner (Fig. 1A and Fig. S1), and this effect was partially achieved through promoting HOXB13 mRNA and protein production (Fig. 1C and S2, S3). Contrarily, a recent report described that knockdown of endogenous HOXB13 by RNA interference in human ovarian cancer cell lines was associated with reduced cell proliferation [36]. These contradictory descriptions may be the results from the apparent difference between ovary and other organs, since HOXB13 is unexpressed in normal ovary [36], whereas it is expressed at a high level in normal prostate and kidney [5], [29]. Nevertheless, data presented in this report provide evidence that ATRA treatment inhibited the growth of DU145 prostate cancer cells, through a mechanism that involved the upregulation of HOXB13 by reducing the methylation level at gene’s promoter.

DNA methylation is catalyzed and maintained by DNMTs. Certain DNMTs, expressed at relatively low levels in somatic cells, are frequently upregulated in cancer cells. Gain-of-function studies showed that DNMT3b but not DNMT3a promoted colon tumorigenesis in APC^Min/+^ mice by inducing *de novo* methylation of multiple genes harboring CpG islands [26]. There was also indication that depletion of DNMT3b resulted in increased apoptosis rate and reduced migration of PC-3 prostate cancer cells [37]. In this study, we showed that HOXB13 expression was silenced in DU145 (Fig. S4), and BSP assays revealed that its silence may be related to the hypermethylation of the gene promoter (Fig. 2A). It was reported that *HOXB13* gene was a target of DNMT3b in colon cancer cells [26]. It therefore becomes important to understand the function of DNMT3b in silencing of *HOXB13* gene in DU145 prostate cancer cells. Our results demonstrated that DNMT3b could regulate *HOXB13* transcription through altering the DNA methylation modification at gene’s promoter, although knockdown of DNMT3b did not reduce the methylation of CpG in *HOXB13* promoter as expected (Fig. 2C, D). Apparently, other mechanisms may exist underlying the DNMT3b-mediated *HOXB13* repression.

In particular, normal and aberrant methylation patterns may be mediated by chromatin proteins guiding DNA methyltransferases to their target genes [38]. Most recently, the PcG protein EZH2 was reported to recruit DNA methyltransferases, especially DNMT1 and DNMT3b, to implement the silencing of the *MYT1* and *WNT1* loci through DNA methylation [21], [32]. Overexpression of EZH2 usually occurs in a diverse of malignancies, including prostate, breast and liver cancers, especially in advanced cases [39]. Varambally *et al* [40] concluded that deregulated expression of EZH2 may be a cause of prostate cancer progression, as well as being a marker that distinguishes indolent prostate cancer from those at risk of lethal progression. Our results suggested that PcGs participated in prostate cancer development. We showed that the expression of EZH2 and BMI1 was much higher in DU145 cells than in LNCap cells (Fig. 3A, B). More interestingly, we found that PcGs suppressed HOXB13 expression in DU145 cells. RT-PCR and western blotting assays showed that HOXB13 expression was upregulated when EZH2 and BMI1 were knocked down (Fig. 3C, D, E and F). Furthermore, our ChIP assays revealed that EZH2 was able to directly bind onto the *HOXB13* promoter (Fig. 4B), and this may be a silencing mechanism of *HOXB13* in the DU145 malignant prostate cancer cells.

It has been proposed that EZH2 represses the transcription of target genes mainly through two different mechanisms. The first involves the binding of EZH2 to the target gene’s promoter to induce the H3K27me3 modification, thereby decreasing the promoter activity [39], [41]. The second mechanism suggests that EZH2 recruits a co-repressor or complex, such as DNMT1 and DNMT3b, to the promoter and this co-repressor either negatively affects other factors that are present, or alters the local chromatin structure to facilitate repression [30], [35]. Apparently, results from this study are in general agreement with both of the two models. Specifically, our data demonstrated that EZH2 was able to directly bind to the *HOXB13* promoter to induce the H3K27me3 modification (Fig. 4B). Meanwhile, our data also suggested that EZH2 may recruit DNMT3b to *HOXB13* promoter to affect the promoter methylation status and hence repress the gene transcription (Fig. 4C, D). These results provide further evidence for a crosstalk between the two distinct epigenetic mechanisms in gene silence.

ATRA is usually used in combination with other drugs, such as docetaxel, TSA and zoledronic acid in prostate cancer therapy [18], [42], [43], but the molecular mechanism of ATRA action is unclear. The fact that EZH2 recruits DNMT3b to the *HOXB13* promoter to repress the gene expression, and that treatment with ATRA results in the upregulation of the HOXB13 expression, intrigued us to propose a hypothesis that ATRA could upregulate HOXB13 through decreasing the methylation level of the gene induced by EZH2 and DNMT3b. Results from this study validated that the expressions of *EZH2* and *DNMT3b* were decreased after ATRA treatment in both DU145 and PC-3 cell lines (Fig.5A, B and Fig. S2, S3). Also, the bindings of EZH2 and DNMT3b onto the *HOXB13* promoter and the H3K27me3 level whereby were reduced in DU145 cells treated with ATRA (Fig. 5C). Interestingly, BSP assays revealed that the DNA methylation level of the *HOXB13* promoter was also decreased in DU145 cells upon ATRA treatment (Fig. 5D).

There are three *RAR* genes, i.e., *RARα, β* and *γ* in cells, and they are all required for ATRA function. Since *RARβ* is silenced in DU145 cells, the sensibility of the DU145 cells to ATRA is lower than that of LNCap cells [44]. Meanwhile, we also examined the effect of ATRA in 293 and 293T cells, the two normal human embryonic kidney cell lines. As expected, the sensibility of these cells to ATRA was higher than that in DU145 cells (Fig. S5A). Noticeably, treatment of AR^−^ prostate cancer cells with ATRA did not change the expressions of HOXB13, EZH2 and DNMT3b at mRNA level (Fig. S5B), implicating a different mechanism of ATRA action in this particular cell background. Presumably, the epigenetic modifications mediated by ATRA described in this report may probably be restricted to certain cancer cell types like AR^−^ prostate cancer cells, since there have been no reports of ATRA-mediated epigenetic modifications in normal cells to date. Likewise, the more malignant and drug resistant PC-3 cells exhibited an even higher sensibility to ATRA than DU145 cells (Fig. S1).

A previous report unraveled that reduced DNMT3b expression resulted in hypomethylation of *retinoblastoma (Rb)*, *RARβ*, and *adenomatous polyposis coli (APC)* gene promoters [37]. Presumably, downregulation of DNMT3b may partly potentiate the sensibility of DU145 cells to ATRA, and this may establish a positive feedback between the ATRA treatment and the downregulation of DNMT3b to facilitate the HOXB13 expression, and consequently to induce the growth arrest of DU145 cells. In this study, we found that ATRA was able to upregulate the expression of HOXB13 and to induce DU145 cells growth arrest as a result of the reduced methylation level of *HOXB13*. This raises a question of whether a synergistic effect between ATRA and DNMTs inhibitors, e.g. 5-aza-dc, exists. Results from our MTT and real-time PCR experiments supported this assumption, since simultaneous treatment of both ATRA and 5-aza-dc exhibited a much stronger inhibitory effects to DU145 cells than each of the two drugs alone (Fig. S6). Meanwhile, the expression of HOXB13 was upregulated, but both EZH2 and DNMT3b were downregulated markedly upon the treatment of the two drugs (Fig. S7).

**Figure 6:**
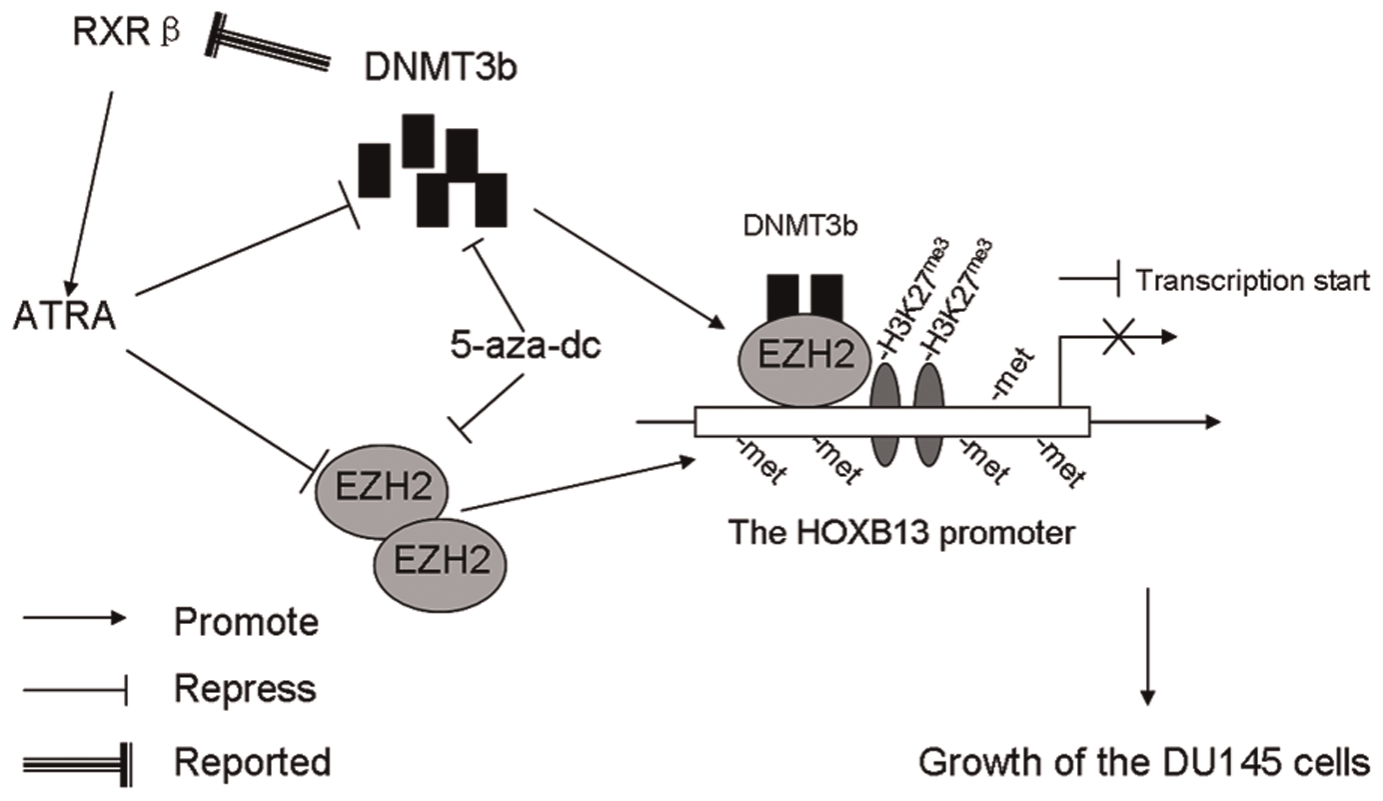
A proposed working model to depict the process of how ATRA induces growth arrest in DU145 cells through upregulating HOXB13. EZH2 directly binds onto the HOXB13 promoter and induces trimethylation of H3K27 to repress the HOXB13 expression. DNMT3b is recruited to HOXB13 promoter by EZH2 to induce the DNA hypermethylation resulting in further repression of the gene. Meanwhile, ATRA represses EZH2 and DNMT3b expression, and impairs their binding at HOXB13 promoter to decrease the methylation level of HOXB13 promoter, resulting in the activation of HOXB13, which in turn inhibits the growth of DU145 cells. Moreover, repression of DNMT3b also upregulates the *RARβ* expression, which may enhance the sensibility of the DU145 cells to ATRA. A combined treatment of both ATRA and 5-aza-dc brings about enhanced effects on HOXB13 upregulation and the inhibition of the growth of DU145 cells.

Altogether, data presented in this report support a working model in which HOXB13, EZH2 and DNMT3b are involved in ATRA-induced cell growth arrest in DU145 cells (Fig. 6). Specifically, ATRA inhibits the proliferation of DU145 prostate cancer cells through reducing the methylation level of *HOXB13* promoter induced by EZH2 and DNMT3b. Perspectively, data arising from this study may provide useful clues for the development of new therapeutic strategies for AR^−^ prostate cancer that involve the use of ATRA and epigenetic modifiers.

## Materials and Methods

### Cell Culture and Reagents

DU145, PC-3, LNCap, 293 and 293T cells were purchased from the Institute of Cell Biology (Shanghai, China). Cells were maintained in F12 (DU145 and PC-3), 1640 (LNCap) and DMEM (293 and 293T) media (Gibco), respectively, supplemented with 10% FBS, 100 µg/ml penicillin and 100 µg/ml streptomycin in a humidified atmosphere containing 5% CO_2_ at 37°C. ATRA (Sigma, R2625-1G) was dissolved in absolute ethanol at 10^–2^ M, and stored at –20°C for in vitro studies. 5-aza-2-deoxycytidine (5-aza-dC, Sigma, A3656-5 mg) was dissolved in acetic acid: water (1∶1) at 10^–2^ M, and stored at –20°C for in vitro studies.

### Transfection and Plasmid Constructs

Transient transfection of DU145 cells was performed using the Fu GENE HD transfection (Roche) procedures. The plasmids for RNA interfering experiments were generated by inserting an oligonucleotide containing a specific siRNA targeting sequence into the pSilencer4.1-CMV-neo vector (Ambion Applied Biosystems, USA). The targeting sequences were: *EZH2*: 5′-cagctctagacaacaaacc-3′ [45]; *BMI1*: 5′-gaccagaccactactgaat-3′ [45]; *DNMT3b*: 5′-agatgacggatgcctagaa-3′ [46] and *HOXB13*: 5′-gaacagcgctacccttaa-3′ [10].

### RT-PCR and Real-time PCR

RNA extraction and RT-PCR were performed as previously described [47]. Quantitative real-time RT-PCR was carried out on an ABI Prism7000 Sequence Detection System (Applied Biosystems), and SYBR Green (TOYOBO) was used as a double-stranded DNA-specific fluorescent dye. The PCR primer sequences were : EZH2, 5′-tctattgctggcaccatctg-3′ (sense), 5′-tgcatccaccacaaaatcat-3′ (antisense); BMI1, 5′-ccagcagcaatgactgtgat-3′ (sense), 5′-ccagcatttgtcagtccatc-3′ (antisense); DNMT3b, 5′-gagtccattgctgttggaaccg-3′ (sense), 5′-atgtccctcttgtcgccaacct-3′ (antisense); HOXB13, 5′-gagccgccaaagcaat-3′ (sense), 5′-acaggcaacagggagtca-3′ (antisense); and β-actin, 5′-tcgtgcgtgacattaaggag-3′ (sense), 5′-atgccagggtacatggtggt-3′ (antisense).

### Western Blot and Co-immunoprecipitation (CoIP)

Protein extraction and western blot analysis were performed in DU145 and LNCap cells as previously described [11]. β-actin was used as an internal control for normalizing the loading materials. Co-precipitation was performed in DU145 cells were described previously [48]. Total cell extracts were pre-cleared with 40 µl protein A-agarose at 4°C for 1 h. The supernatant was incubated with the anti-EZH2 or anti-DNMT3b with gentle shacking for 1 h at 4°C followed by addition of 40 µl of protein A-agarose for another 3 h. The beads were resuspended in 100 µl of 2×loading buffer and boiled for 10 min. The proteins prepared were immunoblotted with anti-DNMT3b or anti-EZH2 antibody. The antibodies used were anti-HOXB13 (Santa Cruz, sc-28333; 1∶250), anti-EZH2 (CST 3147; 1∶1000), anti-DNMT3b (CST 2161; 1∶1000), anti-BMI1 (Upstate Biotechnology 05637; 1∶1000), and anti-β-actin (Sigma, A1978; 1∶6000).

### Chromatin Immunoprecipitation (ChIP)

The protocol for ChIP was described previously [49]. Briefly, the chromatin solution was pre-cleared with 50 µl of protein A/G-agarose beads (Upstate Biotechnology). The soluble fraction was collected and 5 µg of mouse anti-EZH2, rabbit anti-DNMT3b or rabbit anti-H3K27me3 (CST 9733; 1∶1000) antibodies were added. The precipitated DNA fragments were analyzed by PCR. Total chromatin served as the input loading control. The sequences of the primers used were described previously [11].

### Cell Proliferation Assays

The 3-(4, 5-dimethylthiazol-2-yl)-2, 5-diphenyl-2H-tetrazoliumbromide (MTT) assays was conducted to measure cell proliferation. DU145 cells were seeded in 96-well plates at 5×10^3^ cells/well. Cells were transfected using the Fu GENE HD transfection reagent. 24 h later, cells were treated with ATRA at appropriate concentrations. Twenty microliters of MTT (5 mg/ml) was added 24, 48 or 72 h after ATRA treatment. After incubation at 37°C for 4 h, the supernatant was discarded, and 100 µl DMSO was added to each well. Absorbance at 492 nm was measured on a microplate reader. Assays were performed in sextuplicate.

### Extraction of Genomic DNA and Analysis of DNA Methylation

Genomic DNA was extracted from cells using the UNIQ-10 Column Animal Genomic DNA Isolation Kit (Sangon, SK1205). Genomic DNA (1 µg) was modified with sodium bisulfite using the EpiTect Bisulfite kit (Qiagen). The fragment covering 20 CpG sites from HOXB13 promoter region was amplified from bisulfite-modified DNA. The primers used were: forward 5′-agagagagagagagaataagt-3′ and reverse 5′-ccttaactccatccaaaataac-3′ [29]. Amplified bisulfite-sequencing PCR products were cloned into pMD18-T-simple vector (Takara). Sequence analysis was performed by Invitrogen.

### Statistical Analysis

The independent Student’s *t*-test was used to compare the continuous variables between two groups. The level of statistical significance was set at 0.05 or 0.01 for all tests. Results are shown as means±S.D.

## Supporting Information

Figure S1 **ATRA induced PC-3 cell growth arrest in a dose-dependent fashion.** PC-3 cells were treated with various concentrations of ATRA for 72 h and the proliferation rate was assessed by MTT assays. *P<0.05, **P<0.01 (n = 6).
(TIF)

Figure S2 **ATRA affected the expressions of HOXB13, EZH2 and DNMT3b in PC-3 cell line.** ATRA upregulated HOXB13 expression and reduced the expressions of EZH2 and DNMT3b at mRNA (**A**) and protein (**B**) levels in another prostate cancer cells, PC-3.
(TIF)

Figure S3 **A time course of ATRA effect on the expressions of HOXB13, EZH2 and DNMT3b in DU145 cells.** RT-PCR was used to test the changes of the expressions of HOXB13, EZH2 and DNMT3b at mRNA level in DU145 cells treated daily upon 80 µM ATRA.
(TIF)

Figure S4 **The expression of HOXB13 in different malignancy prostate cancer cell lines, DU145 and LNCap.** RT-PCR was used to assess the HOXB13 expression at mRNA level in DU145 and LNCap cells (**A**), and western blotting was used to determine the HOXB13 expression at protein level in DU145 and LNCap cells (**B**).
(TIF)

Figure S5 **ATRA did not alter the expressions of HOXB13, EZH2 and DNMT3b at mRNA level in normal cells, 293 and 293T.** ATRA induced growth arrest in 293 and 293T cells in a dose-dependent manner (**A**). 293 and 293T cells were treated with various concentrations of ATRA for 72 h and tested by MTT assays. *P<0.05, **P<0.01 (n = 6). RT-PCR was used to examine the expressions of HOXB13, EZH2 and DNMT3b at mRNA level in 293 and 293T cells treatment upon 40 µM ATRA for 3 days (**B**).
(TIF)

Figure S6 **The anti-proliferative effect of the combined use of ATRA and 5-aza-dc in DU145 cells.** Cytotoxicity was determined by the MTT assays in 72-hour cultures. Results are the means of six independent experiments. The error bars represent the standard deviations. The concentrations of the two drugs are both in µM. *P<0.05, **P<0.01 (n = 6).
(TIF)

Figure S7 **Coordinate regulation of the expressions of HOXB13, EZH2 and DNMT3b by ATRA and 5-aza-dc in DU145 cells.** The real-time PCR assessments of the expressions of HOXB13 (**A**), EZH2 (**B**) and DNMT3b (**C**) at mRNA level in DU145 cells treated with 5-aza-dc alone, ATRA alone or with a combination of both. *P<0.05, **P<0.01 (n = 3).
(TIF)
