# Supplementary figures and images for: ATRA Inhibits the Proliferation of DU145 Prostate Cancer Cells through Reducing the Methylation Level of HOXB13 Gene
Source: PLoS One. 2012 Jul 13;7(7):e40943. doi: 10.1371/journal.pone.0040943 (PMC3396626; doi:10.1371/journal.pone.0040943)

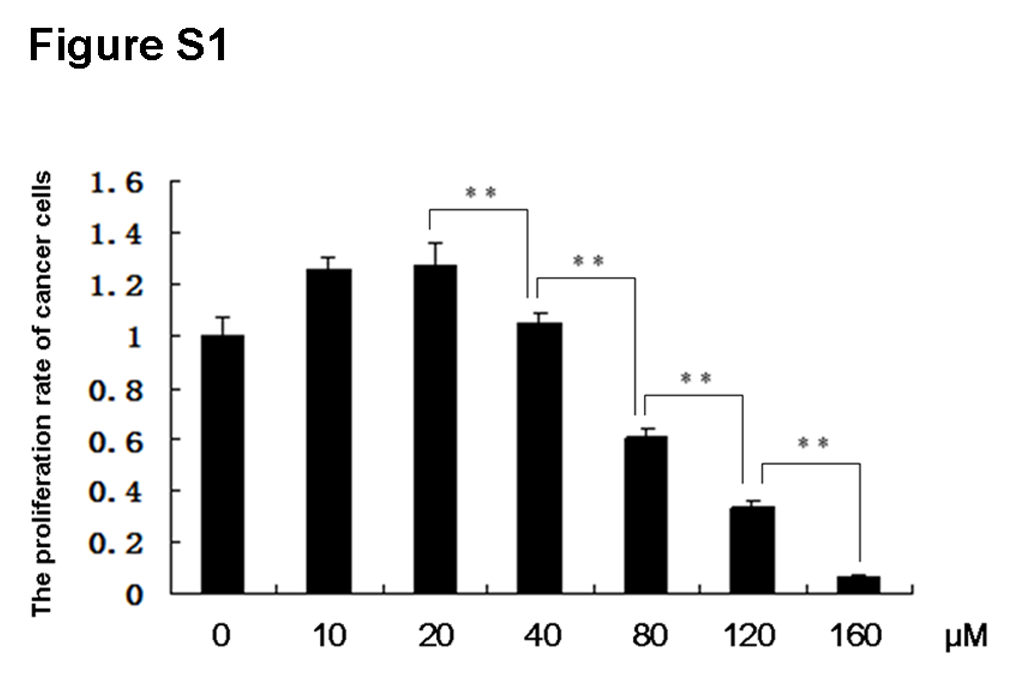

Supplement: Figure S1 — ATRA induced PC-3 cell growth arrest in a dose-dependent fashion. PC-3 cells were treated with various concentrations of ATRA for 72 h and the proliferation rate was assessed by MTT assays. *P<0.05, **P<0.01 (n = 6). (TIF) [file pone.0040943.s001.tif]

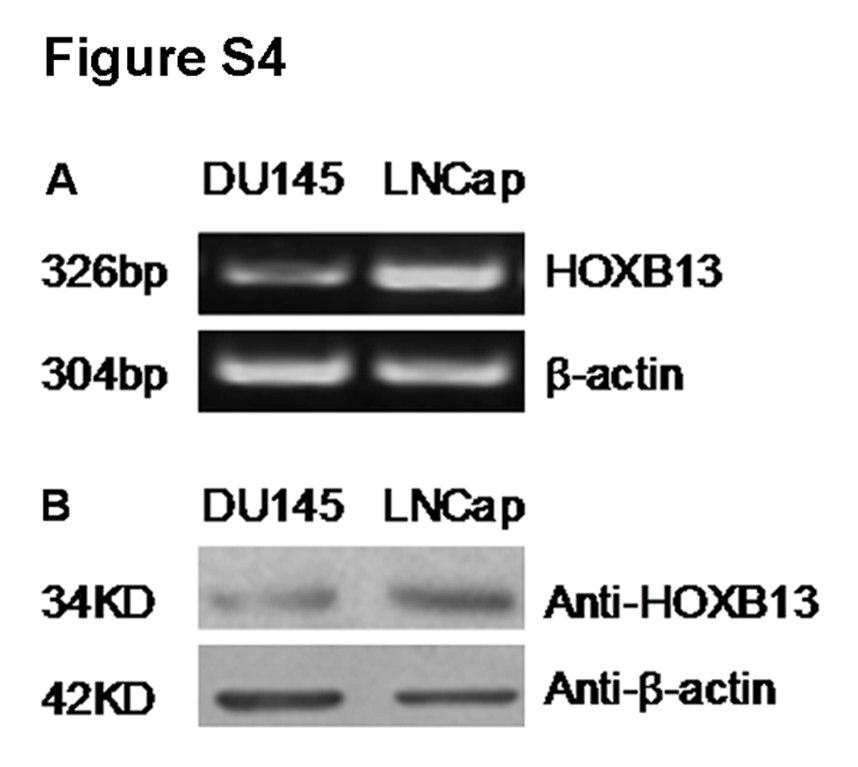

Supplement: Figure S4 — The expression of HOXB13 in different malignancy prostate cancer cell lines, DU145 and LNCap. RT-PCR was used to assess the HOXB13 expression at mRNA level in DU145 and LNCap cells (A), and western blotting was used to determine the HOXB13 expression at protein level in DU145 and LNCap cells (B). (TIF) [file pone.0040943.s004.tif]

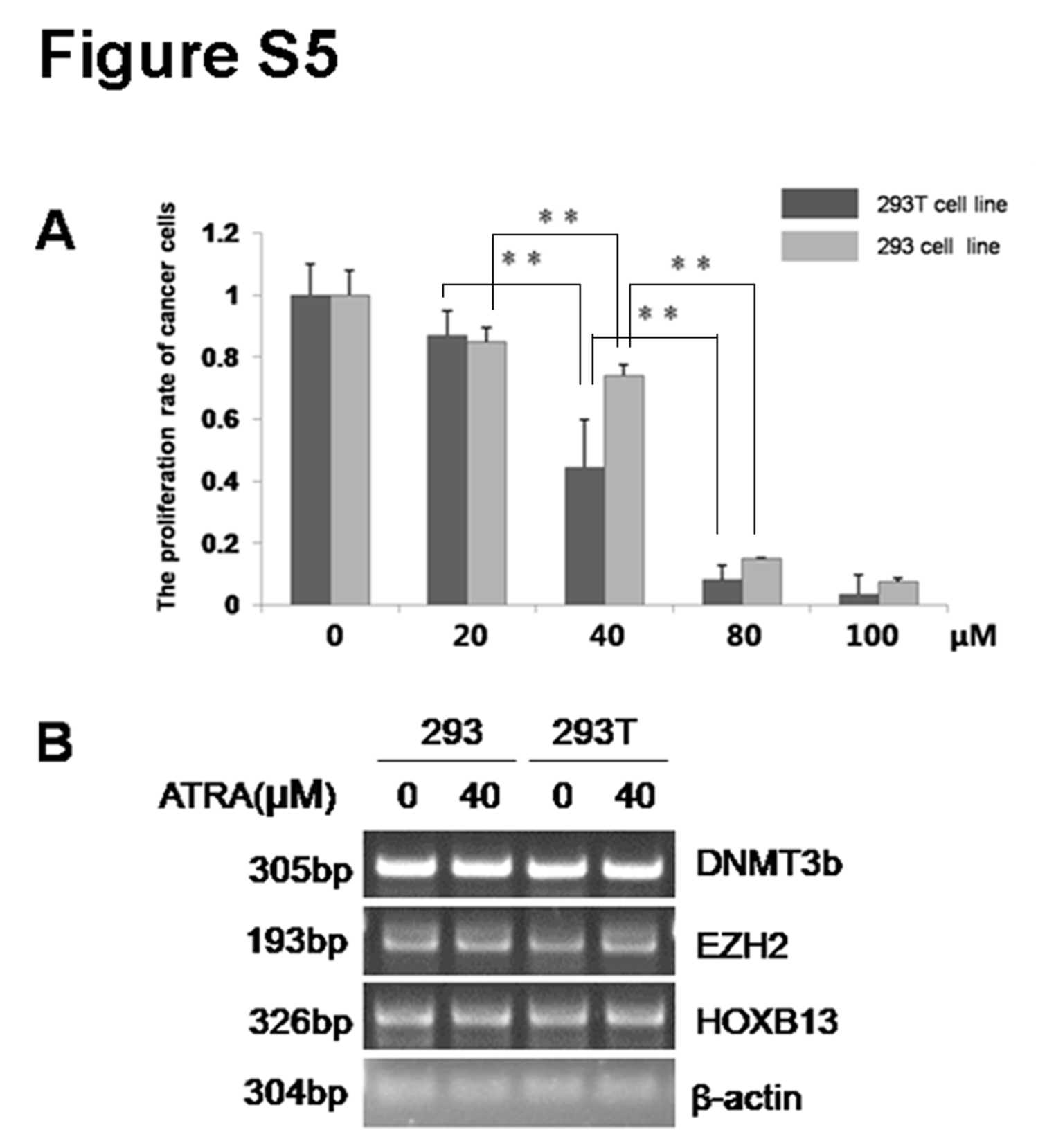

Supplement: Figure S5 — ATRA did not alter the expressions of HOXB13, EZH2 and DNMT3b at mRNA level in normal cells, 293 and 293T. ATRA induced growth arrest in 293 and 293T cells in a dose-dependent manner (A). 293 and 293T cells were treated with various concentrations of ATRA for 72 h and tested by MTT assays. *P<0.05, **P<0.01 (n = 6). RT-PCR was used to examine the expressions of HOXB13, EZH2 and DNMT3b at mRNA level in 293 and 293T cells treatment upon 40 µM ATRA for 3 days (B). (TIF) [file pone.0040943.s005.tif]

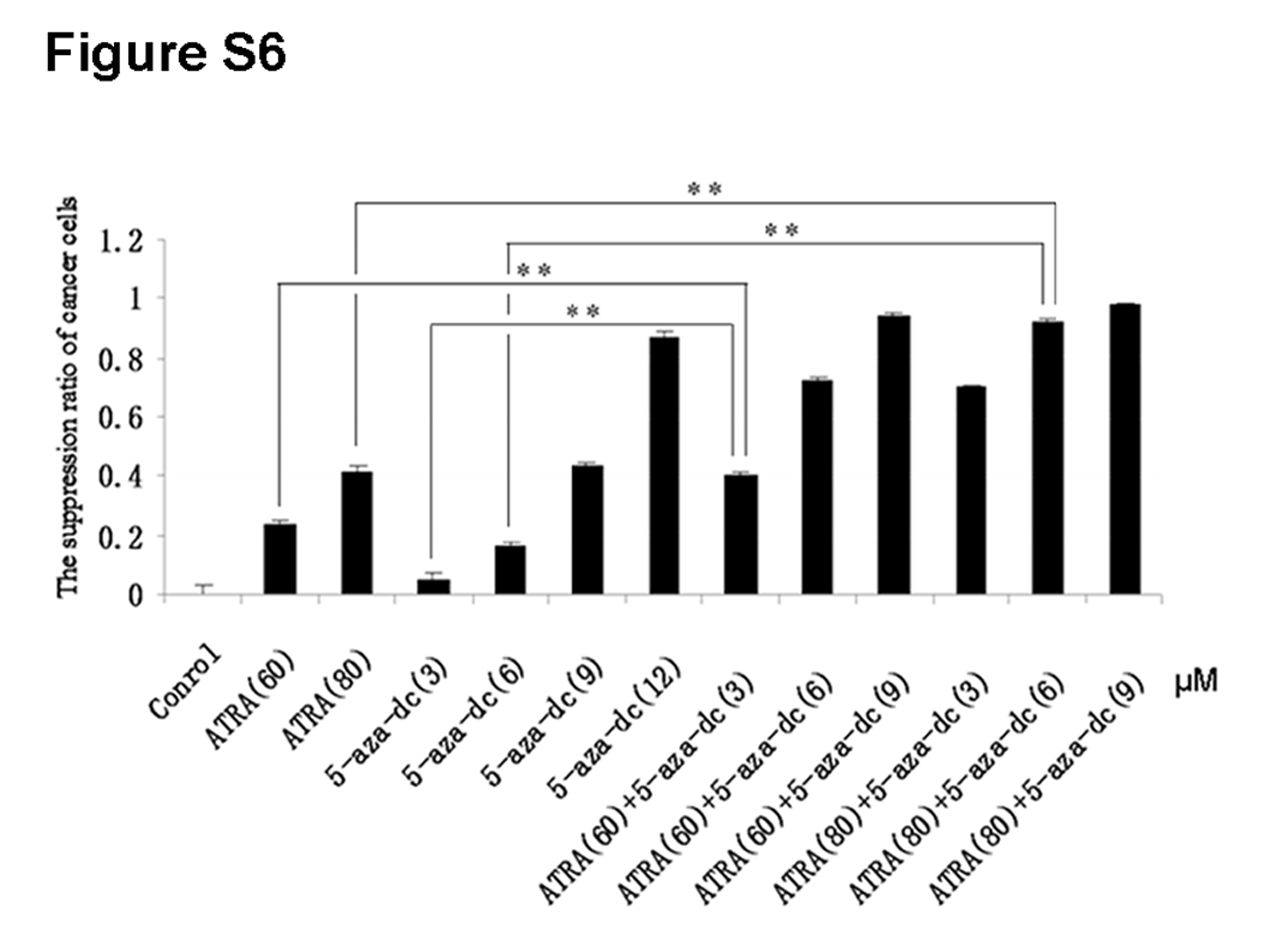

Supplement: Figure S6 — The anti-proliferative effect of the combined use of ATRA and 5-aza-dc in DU145 cells. Cytotoxicity was determined by the MTT assays in 72-hour cultures. Results are the means of six independent experiments. The error bars represent the standard deviations. The concentrations of the two drugs are both in µM. *P<0.05, **P<0.01 (n = 6). (TIF) [file pone.0040943.s006.tif]

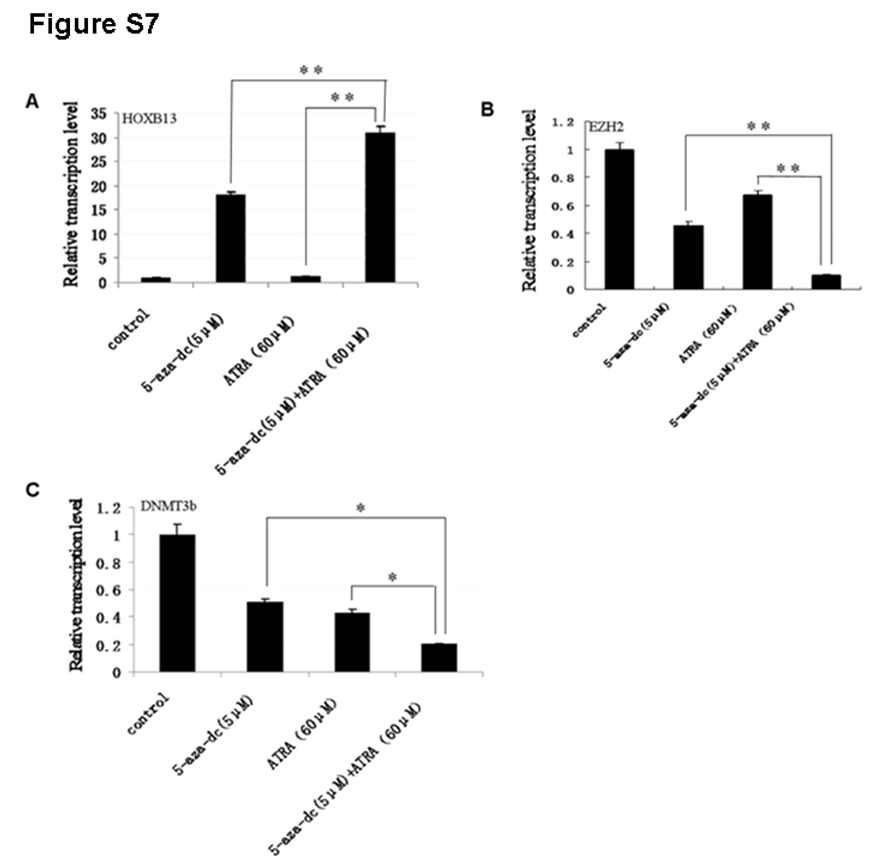

Supplement: Figure S7 — Coordinate regulation of the expressions of HOXB13, EZH2 and DNMT3b by ATRA and 5-aza-dc in DU145 cells. The real-time PCR assessments of the expressions of HOXB13 (A), EZH2 (B) and DNMT3b (C) at mRNA level in DU145 cells treated with 5-aza-dc alone, ATRA alone or with a combination of both. *P<0.05, **P<0.01 (n = 3). (TIF) [file pone.0040943.s007.tif]
